# Lack of Effect of SU1498, an Inhibitor of Vascular Endothelial Growth Factor Receptor-2, in a Transgenic Murine Model of Retinoblastoma

**DOI:** 10.2174/1874364100802010062

**Published:** 2008-04-02

**Authors:** C.M Cebulla, M.E Jockovich, H Boutrid, Y Piña, M Ruggeri, S Jiao, S.K Bhattacharya, W.J Feuer, T.G Murray

**Affiliations:** Bascom Palmer Eye Institute, Department of Ophthalmology, University of Miami Miller School of Medicine, Miami, Florida, USA

## Abstract

SU1498, a tyrosine kinase inhibitor of vascular endothelial growth factor receptor 2 (VEGFR-2), has activity against retinal neovascular diseases. To determine if this drug might have clinical utility against retinoblastoma, we evaluated the effects of SU1498, as well as the expression of VEGFR-2, in a transgenic animal model of retinoblastoma. Optical coherence tomography (OCT) was evaluated as a technology to measure retinal tumors *in vivo*, in response to treatment. Immunofluorescence analysis was performed to evaluate the distribution and expression of VEGFR-2 in enucleated eyes from LHβTag transgenic mice and controls at 4, 8, 12, and 16 weeks of age. VEGFR-2 and phosphorylated (p)VEGFR-2 levels were quantitated by Western blot. OCT was used to pair 10-week-old animals based on tumor volume (n=10), and these animals were treated with 6 periocular injections of SU1498 (50mg/kg, given twice weekly) or vehicle for 3 weeks. Tumor burden was determined by histology and *in vivo* imaging by OCT. VEGFR-2 and pVEGFR-2 expression levels were upregulated during tumorigenesis. However, SU1498 did not significantly reduce tumor burden compared to vehicle (p=0.29). OCT imaging of one matched pair demonstrated equivalent, linear tumor growth despite treatment with SU1498. Retinal tumors can be followed non-invasively and quantitatively measured with OCT. VEGFR-2 is strongly upregulated during tumorigenesis in transgenic retinoblastoma; however, SU1498 does not decrease tumor volume in transgenic murine RB at the studied dose and route of administration.

## INTRODUCTION

Retinoblastoma generates a robust angiogenic response important for its growth and survival [1-5]. Investigating the mechanisms of this response is a major goal for developing new adjuvant therapies for retinoblastoma.

Using the LHβTag transgenic mouse model of retinoblastoma, we have shown that tumor burden is significantly decreased by two independent, anti-angiogenic treatments, combretastatin A-4 [6] and anecortave acetate [7]. Evaluating novel anti-angiogenic agents with different mechanisms of action is a promising strategy, as multiple drugs may eventually be combined for a more robust effect.

VEGFR-2 (also known as KDR or FLK-1) is a high affinity tyrosine kinase receptor for VEGF, known to be very important in mediating normal and pathologic angiogenic responses, especially in cancer [2, 8-14]. Recently, antiangiogenic drugs which inhibit VEGFR have been developed which have shown promise in treating a variety of cancers [15-21]. One promising drug is SU1498, a tyrosine kinase inhibitor specific for VEGFR-2 [22]. Saishin *et al*. previously showed that SU1498 blocks retinal vascular leakage mediated by VEGF in a murine model [23]. With these novel therapies, it is important to confirm the tumor, or its microenvironment, express the molecules targeted by the therapy. Stitt *et al*. demonstrated previously that human retinoblastoma expresses VEGFR-2 [2].

The LHβTag mouse model of retinoblastoma is a transgenic mouse that expresses the SV40 Large T antigen. This protein sequesters p53 and pRB resulting in heritable, bilateral, multifocal retinal tumors that initiate in the inner nuclear layer and are histologically similar to human retinoblastoma [24-29]. Although, the mechanism of tumor initiation between human RB and LHβTag retinal tumors is different, this model is one of the best preclinical models to evaluate novel anti-retinoblastoma therapies. Herein, we tested the hypothesis that VEGFR-2 is upregulated and phosphorylated in transgenic murine RB and that the VEGFR-2 inhibitor SU1498 decreases tumor burden. OCT imaging was used to evaluate the tumor response to therapy *in vivo*.

## MATERIALS AND METHODOLOGY

### Animals

The study protocol was approved by the University of Miami, School of Medicine Animal Care and Use Review Board, Miami, FL. All experiments in this study were conducted in accordance with the Association for Research in Vision and Ophthalmology guidelines for the use of animals in ophthalmologic and vision research. Transgenic mice positive for the LHβTag construct have been previously described, and were identified through polymerase chain reaction analysis of tail DNA [24-29].

### Immunofluorescence Analysis

Transgenic and background control mice were sacrificed with CO_2_ gas at 4, 8, 10-12, or 16 weeks (n=4 per group). Eyes were enucleated, snap frozen in optimal cutting temperature compound (Tissue Tek), and sectioned serially (5 microns). For immunofluorescence, slides were fixed with cold methanol 15 minutes at -20^o^C. Blocking solution (phosphate buffered saline (PBS) with 5% bovine serum albumin, and 1% Triton-X 100) was added to the slides for 1 hour at room temperature and drained off. VEGFR-2 was detected with a rat monoclonal antibody (clone 4H3B6H9, Chemicon International, Temecula, CA) and Alexa Fluor 488-conjugated secondary antibody (Invitrogen). As a control, the primary antibody was omitted. DAPI (Invitrogen) was used as a counterstain. Slides were coverslipped with Antifade mounting media (Biomeda, Foster City, CA).

Twelve to 16 sections per eye were viewed with an Olympus Bx51 fluorescent microscope and images were digitally acquired. Exposure times for VEGFR-2 immunofluorescence were standardized within each experiment.

### Histologic Tumor Burden Analysis

Tumor burden was determined by standard hematoxylin and eosin-(H&E) as previously described [30]. Four of every 6 frozen sections of the eye were fixed in 4% paraformaldehyde and stained with H&E. All stained sections were evaluated for the largest tumor area at a magnification of 40X. Digital photographs were acquired and the tumor area was calculated and normalized to the area of the globe [30].

### Western Blot

Retinas and retinal tumors (n=8 per group) from 4, 8, and 16 week-old LHβTag mice and background controls were removed under a dissecting microscope using a #10 blade scalpel and iris spatula (FST Instruments). Non-retinal structures were removed with jewelers’ forceps. Retinas and tumors were pooled and homogenized with cold extraction buffer (125 mM TrisCl pH 7.0, 100 mM NaCl and 1% Sodium dodecyl sulphate (SDS)). Proteins were quantitated using the Bradford method. Equal microgram quantities of protein were suspended in Laemmli buffer and fractionated with SDS-PAGE on 4-20% precast gels (Invitrogen). Recombinant human VEGFR-2-Fc chimera (R&D Systems) was used as a positive control for VEGFR-2 and pVEGFR-2 proteins. Proteins were electroblotted onto a polyvinylidene fluoride (PVDF) membrane. Membranes were blocked with 5% nonfat milk and incubated with VEGFR-2 primary antibody (FLK-1 (C-1158), Santa Cruz) or pVEGFR2 primary antibody (PhosphoDetect Anti-VEGFR2 (pTyr1214), Calbiochem) and a secondary antibody conjugated to horseradish peroxidase. Blots were developed using ECL chemiluminescence. Blots were stripped and re-probed for β-actin (clone C4, Santa Cruz) as a loading control.

### OCT Analysis

A spectral-domain optical coherence tomography (OCT) system for noninvasive, *in vivo*, high resolution imaging of rodent retina was used as previously described [31]. The configuration of the OCT system used in this study is similar to the previous study except that the depth resolution was improved to ~3 µm in tissue. The right eyes of 10 LHβTag mice were imaged *in vivo*, and the mice were paired according to greatest cross-sectional tumor width: small (<1/3 mm), medium (1/3 mm to <3/4 mm), medium-large (3/4mm to <1mm), large (1mm to <1.5mm), and very large (≥1.5mm). For one select pair (animal numbers II8 and MM8), the tumor location was registered and volume was extracted once a week for 3 weeks (time 0, week 1, week 2, and week 3 post-injection #1). A semiautomatic segmentation tool for segmenting the tumor boundaries in each OCT cross-sectional image was used. The tumor volume was then calculated automatically using the segmented boundaries.

### SU1498 Experiments

Spectral OCT was used to pair 10-week-old LHβTag mice based on tumor volume (n=10). Paired animals were treated with 6 periocular injections (given in a 10µl volume twice weekly for 3 weeks) of either SU1498 (50mg/kg, LC Labs, n=5) or DMSO vehicle (n=5). Mice were sacrificed 4 days after the final injection. Tumor burden was determined by H&E histology and weekly, *in vivo* imaging by OCT. The dose of SU1498 was based on studies by Saishin *et al*. [23], which demonstrated prevention of retinal leakage from intravitreal injection of VEGF in C57BL/6J mice. A similar study without prior OCT selection was also performed by this laboratory in which SU1498 (50mg/kg) was given *via *oral gavage. In this study, 6 doses of SU1498 (or vehicle) were delivered to 10 week LHβTag mice (n=5 per group) twice weekly for 3 weeks. Mice were enucleated at 16 weeks of age, and eyes were formalin-fixed, paraffin-embedded, sectioned, and tumor burden was analyzed by H&E staining.

## RESULTS

**Fig. (1).**
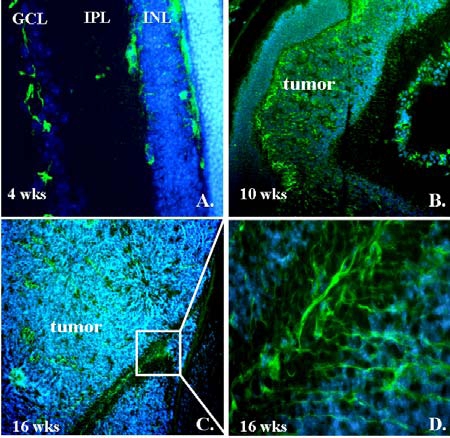
VEGFR-2 immunofluorescence staining in LHβTag transgenic retinoblastoma. Representative eyes from LHβTag mice at 4 (**A**), 10 (**B**), and 16 (**C**,**D**) weeks are shown. VEGFR-2 immunoreactivity is green and DAPI counterstaining is blue. GCL, ganglion cell layer; IPL, inner plexiform layer; INL, inner nuclear; ONL, Outer nuclear layer. Magnification (**A**) 200 X, (**B**) 100 X, (**C**) 200 X. (**D**) zoomed image from inset of **C**.

To test the hypothesis that VEGFR-2 expression is upregulated during tumorigenesis in the LHβTag transgenic model of retinoblastoma, we performed immunofluorescence analyses. LHβTag mice develop multiple, bilateral retinal tumors that are typically not visible on histology at 4 weeks of age, small at 8 weeks, medium–sized at 10-12 weeks, and fill the globe by 16 weeks of age [30]. Eyes from 4, 8, 10-12, and 16 week-old transgenic mice and background controls were evaluated (n=4 per group). In eyes with medium and large tumors, VEGFR-2 expression could be seen in 4 week positive mice in the ganglion cell layer and flanking the inner nuclear layer, but overall levels were very low. In contrast, VEGFR-2 levels were strongly upregulated in 8, 10-12, and 16 week transgenic mice, both in areas of tumor and in the retina (Fig. **1**). VEGFR-2 immunostaining was most intense in the ganglion cell layer, inner plexiform layer, outer plexiform layer, and within the body of tumors. Large tumors (16 week) had VEGFR-2 immunofluorescence throughout the tumor (Fig. **1**). Immunohistochemical analyses suggest that VEGFR-2 is expressed by Muller glia as well as endothelial cells in this tumor. VEGFR-2 staining colocalizes with molecules associated with Muller glia (CRALBP and vimentin) more than with endothelial cells (CD105 and lectin, data not shown).

**Fig. (2).**
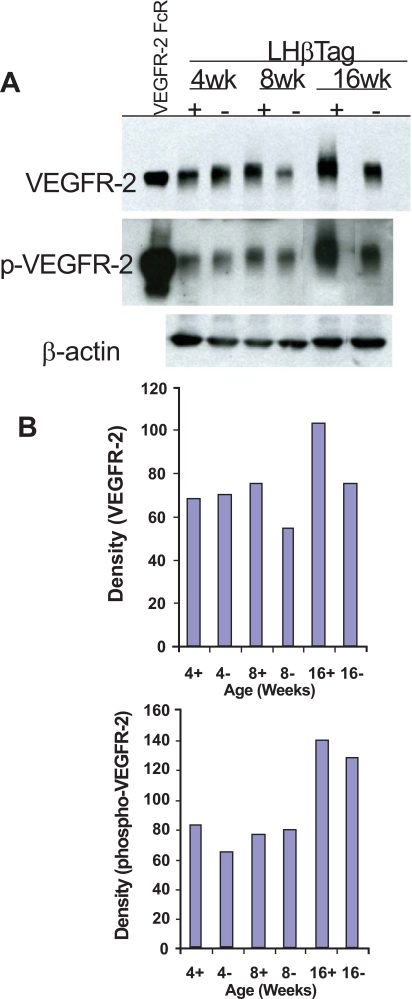
VEGFR-2 and pVEGFR-2 are upregulated in transgenic RB. (**A**) Western blot analysis was performed in retinal/tumor isolates from LHβTag mice and controls at 4, 8, and 16 weeks of age (n=8 pooled retinas per group). Actin was probed as a loading control. One representative blot is shown of 3 replicates. (**B**) Graphic representation of one representative experiment showing quantitative changes in VEGFR-2 and pVEGFR-2 levels (in relative units) from Western blot analysis. Animal age (weeks) and tumor status (+ for positive or – for negative tumor burden) is indicated on the x-axis.

In order to confirm the upregulation of VEGFR-2 during tumorigenesis, we performed Western blot analyses on retinas and tumors isolated from 4, 8, and 16 week LHβTag mice and background controls (n=8 retinas per group). VEGFR-2 levels were elevated in 8 and 16 week old transgenic mice compared with 4 week old mice and negative controls (Fig. **2**). The activation of VEGFR-2 begins with VEGF binding the receptor, leading to its phosphorylation [10]. Western blot analysis of pVEGFR-2 levels was performed to confirm that VEGFR-2 phosphorylation occurs in transgenic retinoblastoma. Levels of pVEGFR-2 were elevated in 4- and 16-week-old LHβTag retinal and tumor extracts compared to controls (Fig. **2**). Immunoblots were probed for β-actin as a loading control (Fig. **2**).

Since VEGFR-2 is upregulated and phosphorylated in transgenic retinoblastoma, we hypothesized that pharmacologically blocking VEGFR-2 would be an effective therapeutic strategy, reducing tumor burden. To test this hypothesis, LHβTag mice (n=5 per group) were treated with SU1498, a drug shown to effectively block retinal leakage in response to VEGF intravitreal injections in mice [23]. The same dose (50mg/kg) was used in this study, and the drug was delivered to 10 week transgenic mice *via *6, 10ul periocular injections twice weekly for 3 weeks. The vehicle, DMSO, was used as a control. Mice were first paired based on tumor size, as determined with spectral OCT imaging. The tumors were grouped into small, medium, medium-large, and large sizes as described in the methods (Table **1**). Side-effects of both DMSO and SU1498 included moderately severe orbital fibrosis in all animals. Other side effects included corneal abrasions and ulceration, conjunctival hyperemia, and neovascularization of the cornea in one animal. Abrasions and ulcerations were treated with erythromycin ophthalmic ointment. No infection or corneal perforations occurred.

**Fig. (3).**
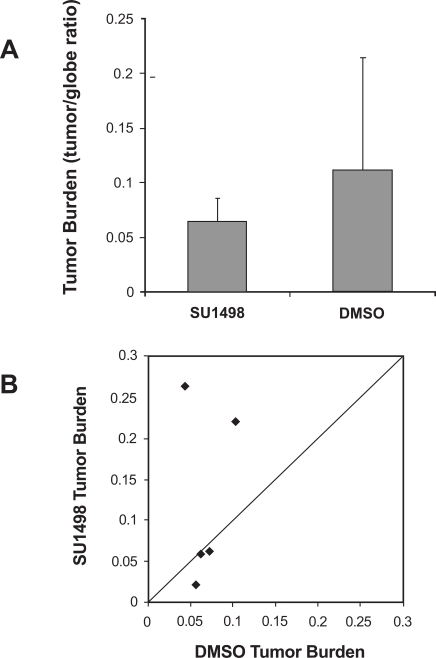
VEGFR-2 inhibitor SU1498 did not significantly reduce tumor burden. (**A**) Histologic analysis demonstrates that the average tumor burden (tumor/globe ratio) of LHβTag right eyes was not significantly decreased by six periocular injections of SU1498, compared with DMSO control. (**B**) Scatter plot of tumor burden in DMSO-treated versus SU1498-treated eyes. Each point represents the tumor burden of one pair, with DMSO tumor burden on the Y-axis and SU1498 tumor burden on the X-axis. The diagonal line indicates equal tumor burden in the SU1498-treated eyes and DMSO control. Pairs in which SU1498-treated eyes had smaller tumors appear in the upper triangle of the graph.

As shown in Fig. (**3A**), the tumor burden was decreased after treatment with SU1498 compared with DMSO control, but not significantly (p=0.29, paired t-test). A 95% confidence interval around the non-significant mean difference of DMSO – SU1498 tumor to globe ratio (0.06) ranged from - 0.08 to +0.19. Similarly, analysis of each pair shows no significant improvement in SU1498 treated eyes compared to controls, although in two pairs the tumor burden was substantially less in SU1498-treated eyes (Fig. **3B**). These negative data were corroborated by another study in which SU1498 (50mg/kg) was delivered to LHβTag mice *via *oral gavage instead of periocular injection (n=5 SU1498 and 5 vehicle controls (data not shown)).

**Fig. (4).**
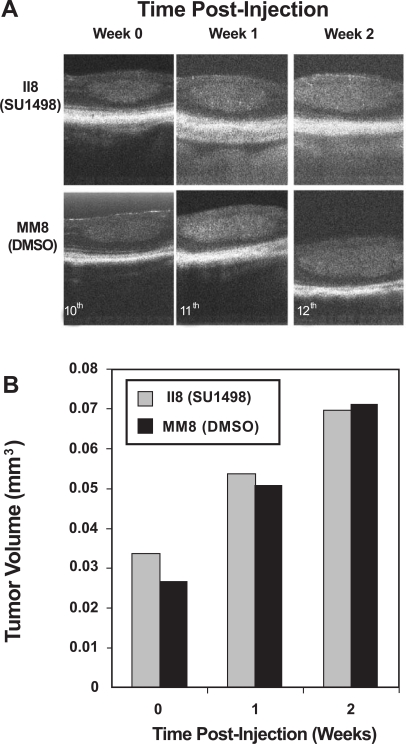
OCT effectively imaged tumor burden changes in response to SU1498 treatment. *In vivo* imaging was performed on a registered tumor in one matched pair of LHβTag mice. (**A**) Pictures of the tumor in cross-section are shown prior to treatment (time 0, age 10 weeks), 1 week post-injection number 1 (11 weeks), and 2 weeks post injection number 1 (12 weeks). (**B**) Graph of the tumor volume determined by OCT segmentation methods.

Spectral OCT technology has now enabled non-contact, *in vivo* imaging of LHβTag retinal tumor response to drug therapies. Two paired mice (study number II8 and MM8) were imaged once each week during the course of the experiment, and their tumors were followed. Both SU1498 and DMSO treated animals showed linear increases in tumor volume during the 2 weeks evaluated, with no significant differences (Fig. **4**). By the third week of the experiment (age 13 weeks), the tumor volume was not measured since tumor size exceeded the detection boundaries of this system (data not shown). The calculated tumor volumes (in cubic millimeters) are shown in Table **2**.

## DISCUSSION

Herein we show that although VEGFR-2 is upregulated and phosphorylated in transgenic murine retinoblastoma during tumorigenesis, treatment with the VEGFR-2 blocking drug SU1498 does not significantly decrease tumor burden at the dose studied, even though SU1498 tumor burden was substantially less in two animal pairs. To our knowledge, this is the first study to (1) pair animals with equivalent ocular tumor burden, in a transgenic model, and (2) follow tumor burden response to drug therapy *in vivo*, with OCT technology.

The dose of SU1498 was carefully selected (a dose with known *in vivo* activity in mice [23]) and was delivered by both periocular injection and oral gavage. In contrast, two other anti-angiogenic drugs: anecortave acetate and combretastatin A-4, both significantly impacted transgenic retinoblastoma tumor volume [6,7].

Of VEGF receptors, VEGFR-2 is most important in mediating angiogenesis; alone it is sufficient to mediate all of the angiogenic responses to VEGF [19,32]. Tumor growth has been successfully inhibited by manipulating VEGFR-2 activity, by using a dominant negative mutant of VEGFR-2 [15] and antibodies that block VEGF activity [20,21]. Further, novel anti-tyrosine kinase drugs specific for VEGF receptors, including PTK787/ZK 222584, ZD 6474, and SU5416, have shown promise in animal models and phase I/II trials for several cancers [16-18,21,33]. However, tumors may be able to make several different angiogenic factors as they develop; allowing compensation for the inactivation of one pro-angiogenic pathway (e.g., tumor bFGF levels increased in breast tumors when VEGF expression was eliminated) [34]. Thus, adding an anti-angiogenic drug with a different mechanism (such as endostatin) to VEGFR-2 inhibitors may significantly improve efficacy, as shown by Abdollahi *et al*. [35].

Anecortave acetate, an angiostatic cortisene, blocks angiogenesis by inhibiting matrix metalloproteinases and urokinase plasminogen activator [36]. Combretastatin A-4 is a microtubule-binding agent which targets angiogenic vessels, in part by disrupting vascular endothelial-cadherin signaling [37]. It is tempting to speculate that the mechanisms of these drugs are more far-reaching in inhibiting the multifaceted angiogenic responses of transgenic retinoblastoma tumors. In fact, inhibiting multiple classes of VEGF receptors or all VEGF isoforms may have a greater efficacy against transgenic retinoblastoma tumors. Recently, Gille *et al*. [38] showed no significant impact on metastatic melanoma tumor burden when just VEGFR-1 or -2 was targeted, but a significant reduction in tumor burden was achieved when both treatments were combined.

In summary, targeting the angiogenic response of retinoblastoma tumors seems to be a promising therapeutic strategy. However, significant testing of combination strategies may be necessary to find the most efficacious therapies.

## Figures and Tables

**Table 1.** LHβTag Mice were Paired into Groups with Equivalent Tumor Burden Prior to Initiating Therapy. The Final Tumor Areas (in Pixels, Determined from Histology) After Treatment are Shown

Pair Number	Initial Tumor Size	Final Tumor Area (SU1498)	Final Tumor Area (DMSO)
1 (II5:LL3)	Very Large	137,509	169,258
2 (II6: JJ9)	Large	69,321	22,721
3 (LL1-LL2)	Large	54,585	324,610
4 (II8:MM8)	Medium	74,099	78,967
5 (KK1:KK3)	Medium	85,129	67,897
**Average**		**84,129**	**132,691**
**Standard dev.**		**31,793**	**119,737**

**Table 2.** Tumor Volume Calculations, in Cubic Millimeters, from Spectral OCT Imaging

Pair Number	Tumor Volume Week 0	Tumor Volume Week 1	Tumor Volume Week 2
II8 (SU1498)	0.0338	0.0540	0.0697
MM8 (DMSO)	0.0267	0.0507	0.0712
